# Lead chromate detected as a source of atmospheric Pb and Cr (VI) pollution

**DOI:** 10.1038/srep36088

**Published:** 2016-10-25

**Authors:** Pyeong-Koo Lee, Soonyoung Yu, Hye Jung Chang, Hye Young Cho, Min-Ju Kang, Byung-Gon Chae

**Affiliations:** 1 Geologic Environment Division, Korea Institute of Geoscience and Mineral Resources, 30 Kajung-dong, Yusung-gu, Daejeon, 305-350, Korea; 2 Korea CO2 Storage Environmental Management Research Center, Korea University, Seoul, 02841, Korea; 3 Advanced Analysis Center, Korea Institute of Science and Technology, 5 Hwarang-ro 14-gil, Seongbuk-gu, Seoul, 136-791, Korea; 4 Department of soil and Groundwater, Korea Environment Corporation, 42 Hwangyeong-Ro, Seo-gu, Incheon 404-708, Korea

## Abstract

Spherical black carbon aggregates were frequently observed in dust dry deposition in Daejeon, Korea. They were tens of micrometers in diameter and presented a mixture of black carbon and several mineral phases. Transmission electron microscopy (TEM) observations with energy-dispersive X-ray spectroscopy (EDS) and selected area diffraction pattern (SADP) analyses confirmed that the aggregates were compact and included significant amounts of lead chromate (PbCrO_4_). The compositions and morphologies of the nanosized lead chromate particles suggest that they probably originated from traffic paint used in roads and were combined as discrete minerals with black carbon. Based on Pb isotope analysis and air-mass backward trajectories, the dust in Daejeon received a considerable input of anthropogenic pollutants from heavily industrialized Chinese cities, which implies that long-range transported aerosols containing PbCrO_4_ were a possible source of the lead and hexavalent chromium levels in East Asia. Lead chromate should be considered to be a source of global atmospheric Pb and Cr(VI) pollution, especially given its toxicity.

## Atmospheric lead and chromium pollution in East Asia

**Figure 1:**
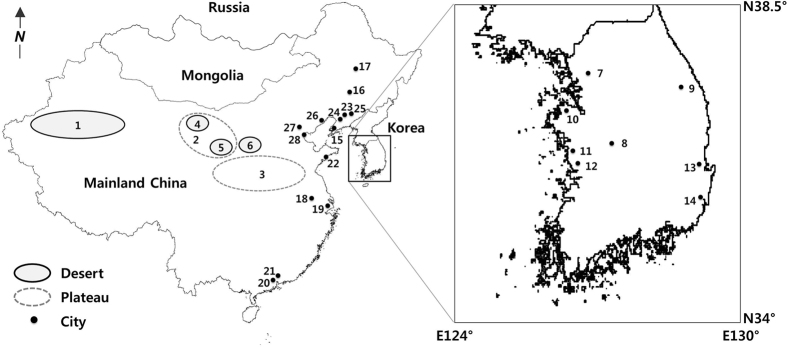
Study area (Deajeon (8); 36°20′N and 127°22′E) and surrounding environment (not to scale; modified after Lee and Yu^26^). The solid box enlarges the South Korea. (1) Taklamakan desert; (2) Alashan Plateau; (3) Loess Plateau; (4) Badain Jaran desert; (5) Tengger desert; (6) Ordos desert are the Chinese deserts soils. (7) Seoul; (8) Daejeon; (9) Taeback; (10) Dangjin; (11) Boryeong; (12) Seocheon; (13) Pohang; (14) Ulsan are the cities in Korea. (15) Dalian; (16) Changchun; (17) Harbin; (18) Nanjing; (19) Shanghai; (20) Foshan; (21) Guanzhou; (22) Qingdao; (23) Shenyang; (24) Anshan; (25) Fushun, (26) Jinzhou; (27) Beijing; (28) Tianjin are the cities in Mainland China.

High levels of Pb and Cr and their seasonal variations are detected in the air in Korea^1^,^2^, China^3^,^4^,^5^ and Japan^6^. In the Korean city of Daejeon (Fig. 1), the arithmetic mean concentrations of lead in Asian dust (AD) and non-Asian dust (NAD) samples were 1,360 μg/g (Total suspended particles; TSP) - 4,560 μg/g (PM_2.5_) and 1,030 μg/g (TSP) - 3,930 μg/g (PM_2.5_), respectively in the year 2007^7^. The mean concentration of chromium in AD and NAD samples ranged from 94 μg/g (PM_10_) to 381 μg/g (PM_2.5_) and from 42 μg/g (PM_10_) to 232 μg/g (PM_2.5_), respectively^7^. The lead and chromium levels did not show a distinct annual variation in 2007–2008, but gradually increased from summer to winter and remained at a relatively high level in the spring^1^. Both AD and NAD samples showed high levels of Pb and Cr^1^,^7^. These high levels of lead and chromium in airborne particulates are of great concern due to their negative impacts on air quality and human health. Chronic exposure to hexavalent Cr-containing particles is known to induce lung toxicity and increase the incidence of cancers in the respiratory system^8^. Adverse health effects of lead exposure, particularly in children, are still a major public health concern, even in developed countries.

Part of the high levels of Pb and Cr in Korea is caused by pollution sources in China given the geographic locations (Fig. 1) and the meteorological conditions. In fact, the rapid industrial development and urbanization in China since the late 1970s have contributed massive quantities of anthropogenic pollutants into the atmosphere on a global scale^9^,^10^,^11^,^12^. In particular, when strong spring monsoons carry dust (i.e., AD) from China, long-range transported AD is one of the main sources of atmospheric pollution in East Asia. AD carries heavy metals from China to the Korean peninsula, to Japan, further to the North Pacific Ocean and even to the USA^13^,^14^,^15^. Moreover, the dust passes through heavily industrialized cities in China and combines with a large number of polluted airborne particulates.

Leaded gasoline was traditionally considered as a major source of Pb in the air, and thus in China and Korea, leaded gasoline was banned in the year 2000 and 1993, respectively. Then the contribution of vehicle exhaust fumes to air pollution decreased. Consequently, the atmospheric air quality improved. However, the lead contamination in the air is still severe in Beijing, Shanghai and Tianjin^10^,^16^,^17^,^18^. In fact, in Beijing, Pb levels remarkably increased in 2001 to 2006^4^, while they prominently decreased in Seoul in 1999–2010^2^. Moreover, the blood lead levels of children in China are higher than those in developed countries, and childhood lead poisoning remains a grave public health concern in China^19^. This fact indicates that sources other than leaded gasoline contribute to the airborne lead in China.

A number of previous studies suggest that lead emissions from coal and oil combustion, non-ferrous metallurgic industries, wind-blown soil dust and cement-derived construction dust are the major contributors to the amount of lead in the atmosphere since the use of leaded gasoline was banned^16^,^17^,^18^. In addition, lead-bearing airborne particles in the atmosphere were attributed to the re-suspension of historically deposited emissions from leaded gasoline combustion^20^,^21^,^22^. However, the sources of Pb and Cr(VI) in the aerosols of East Asia are still not clearly defined. Source identification is required to develop effective pollution mitigation strategies.

In this study a total of 10 Pb-Cr-containing particles from 5 dust dry deposition samples collected in Daejeon in 2007–2008 (Table 1) were intensively investigated for the identification of nanosized Pb-Cr-containing minerals, using scanning electron microscope (SEM) and STEM (scanning transmission electron microscope) equipped with energy-dispersive X-ray spectroscopy (EDS), and electron probe micro analyzer-wavelength dispersive spectroscopy (EPMA-WDS). For the identification of mineral phases, selected area diffraction patterns (SADPs) were obtained in TEM (transmission electron microscope). In Table 1, AD samples were collected when the Korean Meteorological Research Institute (METRI) issued an AD warning, while NAD samples were collected in spring and winter to avoid the rainy season^1^,^7^. All the samples were collected at 13.2 m elevation on the roof of a building of the Korea Institute of Geoscience and Mineral Resources (KIGAM).

## Black carbon and lead chromate aggregates in the dust dry deposition

**Figure 2:**
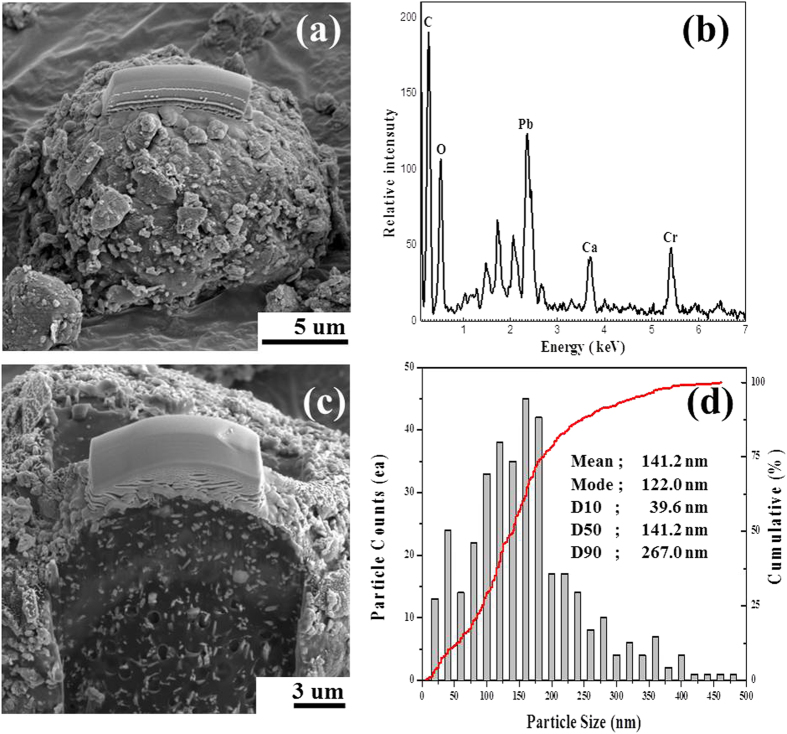
SEM analysis of a Pb-containing particle in a dust dry deposition sample (NAD-7). (**a**) Pb-containing dust particle; (**b**) EDS spectrum of (**a**); (**c**) Cross-sectioned SEM image after ion milling; (**d**) Particle size distribution.

Under SEM, ball-like black carbon aggregates were occasionally observed in the dust dry deposition (Fig. 2a). The size of these spherical aggregates was usually several tens of μm in diameter. Microsized black carbon particles were detected in forms of spherules in the dry deposition of AD and NAD in the study area (Daejeon)^23^ and in the AD samples in Seoul^24^. Kim *et al*.^24^ analyzed a total of 950 individual particles from 30 AD subsamples collected in 2004–2007 under SEM with EDS, and found that carbon was the most abundant element.

This present study investigated about 800 individual particles from 5 dust dry deposition samples (Table 1). Pb was detected in the EDS spectrum of 10 particles as shown in Fig. 2b. The EDS spectrum showed that all the Pb-containing particles comprised Cr (Fig. 2b), similar to the observation in Seoul where Pb and Cr co-occurred in seven individual particles of 950^24^.

The occurrence of Pb-Cr-containing particles was examined in a dust dry deposition sample (NAD-7) by EPMA-WDS elemental mapping (Supplementary Fig. 1). Among thousands of particles within the scanning area of 585 μm × 585 μm (Supplementary Fig. 1a), nine particles were revealed to contain both Pb and Cr in the WDS mapping (Supplementary Fig. 1b,c). According to the chemical composition analysis of WDS spectrum (Supplementary Fig. 1(e)), small amounts of Pb and Cr were determined with high contents of carbon in dust particles.

**Figure 3:**
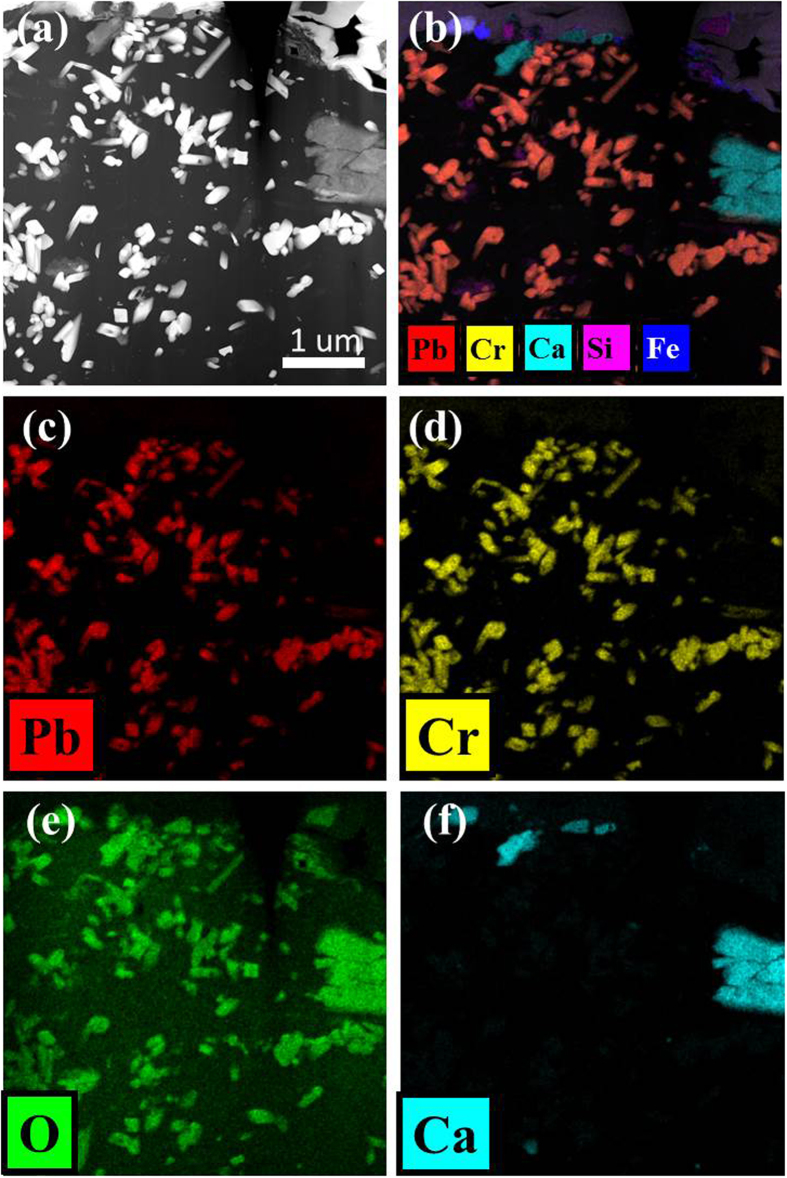
STEM-EDS element map images of a Pb-containing particle in a dust dry deposition sample (NAD-7). (**a**) HAADF STEM image after FIB (focused ion beam) sampling; (**b**) Merged EDS element map; (**c**~**f**) EDS element map images of Pb, Cr, O, and Ca. The Pb-Cr-O phase is predominant within the carbon matrix.

Cross-sectioned SEM and HAADF (high angle annular dark field) STEM images revealed that the carbon aggregates, which included Pb and Cr, were compact and consisted of several mineral phases within a black carbon matrix (Figs 2c and 3a). These mineral phases were present as discrete euhedral to subhedral grains and had needle-, rectangular-, quadrate-, and irregular-shaped morphologies. According to the elemental composition analysis of the individual nanoparticles (Fig. 3b), the minerals within the black carbon matrix could be classified into the following groups: mainly Pb-Cr-O and trivially Ca-C-O, Al-Si-O and Fe-O.

The elemental maps in Fig. 3c–e show that the nanosized particles that bore Pb, Cr and O were coincident with each other and corresponded to a bright contrast in the black carbon matrix of the HAADF STEM image (Fig. 3a). Minerals associated with Ca, Al, Si and Fe were characterized as calcite, silicate minerals and Fe-oxides, and represented a trivial amount within the black carbon aggregate. In addition, a variety of submicron-sized mineral particles, such as rutile, quartz, iron oxides, calcite and clay minerals, adhered to the surface of the spherules of black carbon aggregates (Supplementary Fig. 2).

**Figure 4:**
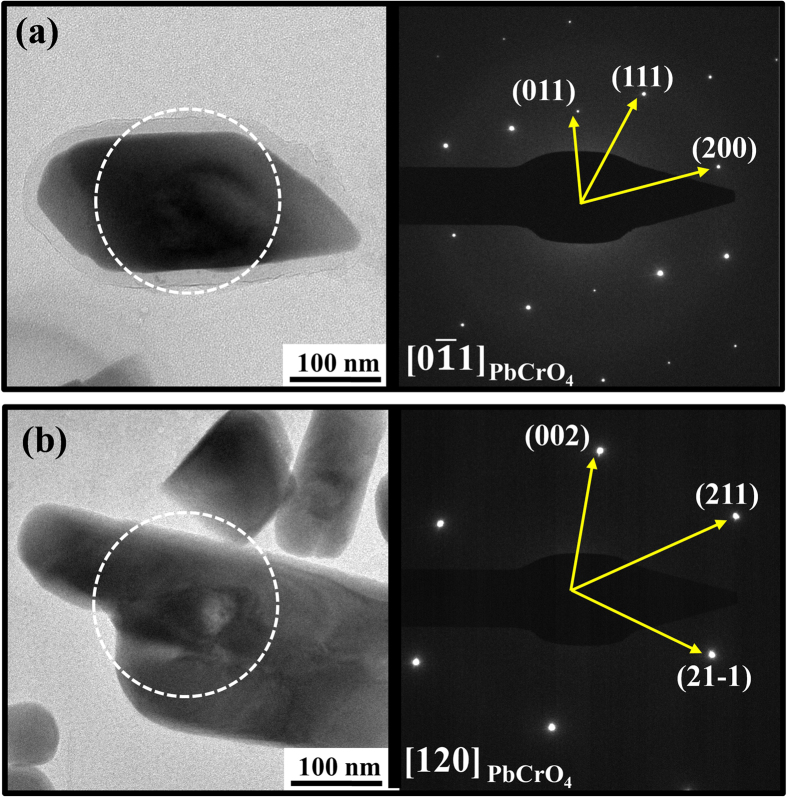
PbCrO_4_ of Sys.: Monoclinic, S.G.: P21/n (167). (**a**) Bright-field TEM image of PbCrO_4_ nanoparticles in carbon matrix and corresponding SADP from [0
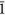
1]; (**b**) Bright-field TEM image of PbCrO_4_ nanoparticles in carbon matrix and corresponding SADP from [120].

In order to identify the predominant Pb-Cr-O phase within the black carbon matrix, the particle sample was characterized by SADPs. The SADPs obtained from the nanoparticles were indexed as the [120] and [011] zone axis of the monoclinic lead chromate (PbCrO_4_), which belongs to a space group of P21/n (167) (Fig. 4). In fact, Lee *et al*.^23^ also observed nanosized mineral phases with heavy metals, including Pb-Cr-O, within the spherical black carbon, but failed to identify PbCrO_4_. In addition, Kim *et al*.^24^ detected seven Pb- and Cr- containing particles of a total of 950 individual particles. However, Kim *et al*.^24^ did not investigate the internal structure of the spherule, and detected Pb and Cr on the surface of the seven carbon-rich spherical particles.

For the quantitative measurement of particle sizes, the HAADF STEM image was statistically analyzed. Since all the prepared 10 TEM samples showed the similar morphology, one typical image at low magnification was chosen for the image analysis. In result, the size of the lead chromate particles ranged from <25 nm to 700 nm in length (mean = 141 nm), and 90% of the lead chromate particles were less than 267 nm in length (Fig. 2d). The areal fraction of lead chromate was expected to be approximately 2.5% in the carbon aggregates.

## Other minerals in the dust dry deposition

In addition, there were black carbon aggregates containing round shaped particles in the dust dry deposition (e.g., Supplementary Fig. 3). An STEM-EDS spectrum confirmed that the round shaped nanosized particles were mostly 200–300 nm TiO_2_ and trivially Ca-Mg-O, Si-O, Fe-O and Si-Al-O (Supplementary Fig. 4). SADP analyses (Supplementary Fig. 5) indicated that the particles were rutile (TiO_2_; tetragonal, P42/mnm(136)), dolomite (CaMg(CO_3_)_2_; hexagonal, R-3(148)), quick lime (CaO; cubic, Fm-3m(225)), and quartz (SiO_2_; hexagonal(Primitive)). Ti was detected on the surface of 52 individual particles of 950 in Seoul as well, and Ti (52 of 950) was more frequently detected than Pb and Cr (7 of 950)^24^.

In contrast, the present study did not confirm the presence of PbSO_4_, PbCO_3_ or any other Pb speciation except PbCrO_4_ in the dust dry deposition, similar to Kim *et al*.^24^ who found that sulfur-containing particles did not contain either Pb or Cr, and Pb and Cr co-occurred in seven individual particles. However, PbSO_4_ and PbCO_3_ are reported to be frequently found in urban air^25^. Jian *et al*.^25^ deduced that Pb speciation consists of 61% of Pb sulfate and 39% of Pb carbonate in urban air (Standard Reference Material (SRM) 1649a) based on the results of XANES (X-ray absorption near edge structure) analyses.

According to the sequential extraction analysis for the dust dry deposition in the study area^23^, Pb was predominately associated with carbonate (36% in AD; 42% in NAD) and reducible fractions (32% in AD; 29% in NAD); while Cr was predominately bound to residual fraction (71% in AD; 46% in NAD), and then oxidizable (16% in AD; 29% in NAD) and reducible (12% in AD; 23% in NAD) fractions. The mismatch between SADP (PbCrO_4_ only) and the sequential extraction analysis (Pb bound to carbonate and reducible fractions^23^) implies that the dust dry deposition contained Pb in various forms in addition to the discrete mineral phase (i.e., PbCrO_4_), such as Pb incorporation into the structure of calcite, dolomite or Fe-Mn (oxy)hydroxides. The low detection rate of Pb-containing particles (i.e., 10 of about 800 individual particles in this study; 7 of 950 in Seoul^24^; Supplementary Fig. 1), despite the high levels of Pb in the dust dry deposition^1^,^7^, also implies the presence of Pb in the dust dry deposition in various forms, which cannot be observed under SEM-EDS probably because they were not present in discrete mineral phases. Besides, the speciation of PbCrO_4_ and non-detection of PbCO_3_ and PbSO_4_ within the black carbon matrix by using SADP in this study implies that the sequential extraction analysis is not a perfect tool to determine the speciation.

On the other hand, as for chromium, a common occurrence in ambient atmosphere is chromite^25^. Minor signature of the presence of Cr(VI) was detected in indoor air (SRM 2584), but none in urban air (SRM 1649a)^25^. The Cr-Pb (+Ti) phase detected in indoor air (SRM 2584) was identified as PbCrO_4_. Similarly, this study (Fig. 2b) and Kim *et al*.^24^ showed that Pb and Cr co-occurred, which indicates that lead chromate is a source of both Pb and Cr(VI) in the air.

## Evidence of long-range transport

The previous result of Pb isotopic analysis showed that the mean Pb isotope ratios (^206^Pb/^207^Pb) of AD (1.1508 ± 0.0096)^1^,^7^ and NAD (1.1531 ± 0.0047)^1^,^7^ were significantly lower than those of the regional soil (1.1822 ± 0.0060)^7^, fly ash from coal-fired power plants (1.2110 ± 0.0072 from Seocheon, Boryeong, Dangjin in Fig. 1)^26^ as well as domestic coal (1.2202 ± 0.0035 from Taeback in Fig. 1)^26^ and Pb ores (1.1822 ± 0.0411)^27^ in Korea. Likewise, the values were significantly higher than the calculated ^206^Pb/^207^Pb of Pb ores imported to Korean lead smelters (1.1343)^26^. These differences indicate that the Pb in AD and NAD did not come from emission sources of Korea. Airborne particles in Pohang steel industrial complex (^206^Pb/^207^Pb of 1.145) and flue gas emission in a lead smelter (^206^Pb/^207^Pb from 1.136 to 1.155) in Ulsan in Korea were not considered because of their geographical locations (Fig. 1) and the little possibility to affect the air quality in the study area given the predominant wind directions^26^.

The mean ^206^Pb/^207^Pb ratios of AD and NAD were in agreement with those of airborne particulates from heavily industrialized Chinese cities (1.1594 ± 0.0119 from Shanghai^18^, Dalian^28^, Changchun^28^, Harbin^28^, Nanjing^28^, Foshan^29^, Guanzhou^29^, Beijing^30^), Chinese coal (1.1627 ± 0.010)^17^, coal combustion dust (1.1668 ± 0.002)^17^ and coal fly ash (1.1655 ± 0.002)^17^ from northern China (Shanghai), and Pb ores from southern China (1.1797 ± 0.0074)^31^,^32^,^33^,^34^. This similarity implies the predominant contribution of Chinese airborne particles to the atmospheric pollution in the study area. Furthermore, the average ^206^Pb/^207^Pb ratio of AD and NAD was clearly distinct from that of the particles from vehicle exhaust fumes in China (1.1294 ± 0.0075)^18^.

Besides, air-mass back trajectory analysis using the HYbrid Single-Particle Lagrangian-Integrated Trajectory (HYSPLIT) model showed that air-masses carrying AD were initiated from deserts and semi-arid areas in China, Mongolia or Siberia, and arrived in Korea after traveling through the heavily industrialized Chinese eastern costal or northeastern areas (Fig. 1)^1^,^24^. In addition, rare earth elements (REEs) within the rounded particles in the dust dry deposition in the study area (Daejeon) also support the long-range transport from China, given that China is the world’s largest REE producer^23^. Inverse modeling analysis also implies that soil dusts in Korea originate from China^35^.

It should be noted that Pb and other heavy metals in AD and NAD seem to originate from the same sources, based on the similar Pb isotope ratios of AD and NAD^1^,^7^ and the major mineralogical compositions^23^. For both AD and NAD, ^206^Pb/^207^Pb and ^206^Pb/^204^Pb isotopic compositions in residual fraction of the dry deposition were similar to the mean ^206^Pb/^207^Pb and ^206^Pb/^204^Pb in residual fraction of the Alashan Plateau soil^26^, which indicates that the geogenic materials of the dry deposition of both AD and NAD were largely influenced by the Alashan Plateau soil. In fact, in Korea, northwest winds from China are predominant except for summer, which may cause the high level of Pb in winter in Deajeon^1^ and Seoul^2^. Besides, the particles move to Korea through eastern Chinese cities even in summer^36^.

We acknowledge that the evidence of long-range transported AD and NAD does not support the long-range transport of the black carbon and lead chromate aggregate (Fig. 2) within the dust dry deposition. Besides, the Pb isotope ratios of the dust dry deposition^1^,^7^ did not represent those of PbCrO_4_ because there would be various forms of Pb in the dust dry deposition. The aggregate containing the discrete lead chromate mineral might originate from local sources and mix with the long-range transported dust. Especially, given the high density of lead chromate (6.12 g/cm^3^), there is little possibility that lead chromate travels long distance in the air. However, the black carbon aggregate including lead chromate can travel long distance because the small portion (areal fraction of 2.5%) of PbCrO_4_ within the matrix does not significantly increase the density of black carbon. The evidence of long-range transported dust implies that at least part of the contaminants within the dust travel long distance.

## Source of spherical black carbon and lead chromate aggregates

The microstructure of the dust in Fig. 2 indicates that Pb- and Cr- bearing nanosized particles were combined with black carbon particles to form black carbon aggregates. The morphology (i.e., spherical carbon matrix) and the internal structure (i.e., discrete lead chromate particles) indicate that the aggregates were related to pollutants in roads.

The major component (i.e., black carbons) in the spherical aggregates came from an incomplete combustion of fossil fuels. Vehicles are a major black carbon source^37^,^38^. In particular, their primary spherical particles have the higher surface areas than industrial black carbon’s^39^.

Meanwhile the natural mineral PbCrO_4_ (crocoite) is known to be found restrictively and primarily in Tasmania, and its particle size is much larger than the sub micrometer size^40^. Conversely the commercial pigment lead chromate (chrome yellow) is produced synthetically, and used in paints and printing inks and as a colorant in vinyl, rubber, and paper. Due to the various applications, lead chromate is found in indoor air^25^. However there is little chance that lead chromate in indoor air is coated with black carbon. Moreover, lead chromate is used extensively as the yellow pigment in road markings^41^,^42^. In particular, lead chromate is used for applications that require safety attributes such as high visibility and therefore are used in traffic paint striping for highways and airports, and safety identification paints on buses, ambulances and fire trucks^43^. Based on its primary application as well as the morphology (Fig. 2), the lead chromate within the black carbon matrix probably came from traffic paint.

The lead chromate in road markings can be separated from the yellow traffic paint through abrasion and resurfacing^40^. The nanosized particles can easily be liberated. Indeed, lead chromate has been found in road dust from heavily used roads^44^,^45^. Re-suspended road dust can be one of the major sources of particulate air pollution^20^.

TiO_2_ and CaMg(CO_3_)_2_ that are the primary ingredients of white paint were also frequently observed within the carbon matrix in Daejeon (Supplementary Figs 3, 4 and 5) and Seoul^24^. The black carbon and TiO_2_ aggregates also seem to originate from roads.

We acknowledge that black carbon and PbCrO_4_ may come from indoor combustion of coal and re-suspended soil dust containing old building debris, respectively, given the coal consumption for residential heating and cooking in China. Paint materials (e.g., lead chromate) can be mixed with carbon from coal combustion in China. However, construction materials (e.g., CaO) were little observed in dust samples.

## Mechanism for the formation and long-range transport of spherical black carbon and lead chromate aggregates

Black carbon and lead chromate monomers can be combined to form compact microscale spherical aggregates. Nanoscale carbon spheres generate chain-like aggregates (soot)^39^,^46^,^47^. Then, the particles can be compacted into spheres after trace gas and/or liquid adsorption and evaporation^46^. In addition, when black carbon is coated, black carbon clusters collapse into a more concentrated form and become spherical-like with larger fractal dimensions^48^. Indeed, carbon-rich spherical particles were found in Beijing^10^, Seoul^24^ and Mexico^49^, and generated in the experiment^50^,^51^. During formation and compaction, lead chromate can be internally mixed with black carbons, especially with diesel engine exhaust soot that has high surface areas^39^.

The particle aggregates are subject to turbulence due to wind and temperature fluctuations in the urban surface boundary layer, which causes the microsized particles to rise and disperse^52^. In addition, the vehicle traffic is known to re-suspend particulates deposited on road surfaces, which results in increased pollution levels^20^,^53^. Then, periodic monsoonal winds from northern and north-western China can transport lead chromate - rich black carbon spherules lifted from heavily industrialized Chinese cities to the city of Deajeon in Korea. Indeed, the long-range transport of several micrometer-size airborne particles, such as black carbon particulates, has been observed throughout the world^54^. It is important to note that the inclusion of heavy lead chromate (6.12 g/cm^3^) cannot hinder the long-range transport of aggregates because the portion of lead chromate in black carbon aggregates is relatively small (approximately 2.5% area).

We admit that there are local sources of the black carbon and lead chromate aggregate in Korea, although the inorganic yellow pigments, including PbCrO_4_, were no longer available in road markings in Korea since 2006. For instance, PbCrO_4_ might come from the re-suspension of historically deposited emissions from old road paint or from abrasion and resurfacing of the road makings painted before 2006. However, the effect of long-range transported lead chromate on the air quality will increase unless PbCrO_4_ is banned in road painting in China.

## Environmental significance of the discovery of lead chromate in aerosols

Local chemical and crystal structure analyses using SEM-EDS and STEM-SADP showed that the spherical black carbon aggregates in the dust dry deposition contained nanosized lead chromate particles. The internal matrix of the atmospheric black carbon aggregates and the chemical composition and crystal structure of the individual nanoparticles indicate that PbCrO_4_ was not a secondary product, but that it originated from an anthropogenic source, i.e., traffic road paint. The yellow pigment in traffic paint has not been considered as a major Pb and Cr (VI) source in the atmosphere. It was not until recently that lead chromate from yellow traffic paint began to be considered as a potential source of lead and hexavalent chromium pollution in surface water and sediments of urban environments. Lately, road dust samples were found to have significantly higher Pb and Cr concentrations than coal and fly ash samples in Hamilton, Ohio^55^, which implied that the coal combustion was not the dominant source of Pb and Cr in road dust despite the proximity to a coal-fired power plant.

This research result singled out lead chromate from traffic paint as a source of lead and hexavalent chromium pollution in the atmospheric environment of Korea. The Pb and Cr(VI) contamination of airborne particles by PbCrO_4_ would be a worldwide problem due to its widespread and international use. The levels of Pb and Cr in the atmosphere are still high around the world, even after the banning of leaded gasolines, and lead is persistent in the environment. A proper treatment of lead chromate in the air is needed to control and improve the air quality.

Based on a Pb isotope analysis and air-mass backward trajectory analysis, airborne particles in heavily industrialized Chinese cities are a possible source of pollutants in the dust dry deposition in the study area. This result suggests that the lead chromate in traffic paint from heavily industrialized Chinese cities was a possible source of Pb and Cr (VI) in the atmospheric environment of Korea. This contamination probably affected other countries in East Asia and even possibly the western states of the USA via long-range transport.

The composition, size and morphology of PbCrO_4_ within the long-range transported aerosols imply that the Pb and Cr (VI) atmospheric pollution due to the dispersion of yellow traffic paint should be globally considered. Besides, it should be noted that chronic exposure can be harmful, especially to children who are frequently exposed to yellow and orange paints for safety (e.g., school bus), although the frequency of PbCrO_4_ occurrence was low in this study. Nanosized particles can cause pulmonary and gastrointestinal health impacts on the world population. Furthermore, because lead chromate contains both Pb and Cr (VI), it is profoundly toxic and carcinogenic^56^. Lead chromate is suspected to be a cardiovascular or blood toxicant, immunotoxicant, kidney toxicant, neurotoxicant, respiratory toxicant, and a skin or sense organ toxicant^57^,^58^,^59^,^60^. Indeed, lead chromate has been largely replaced by another pigment, cadmium yellow^40^,^61^.

## Additional Information

**How to cite this article**: Lee, P.-K. *et al*. Lead chromate detected as a source of atmospheric Pb and Cr (VI) pollution. *Sci. Rep.* **6**, 36088; doi: 10.1038/srep36088 (2016).

## Supplementary Material

Supplementary Information

## Figures and Tables

**Table 1:** Sampling date, mass of the dust dry deposition, and mass concentrations of Asian dust (AD) and non-Asian dust (NAD).

Sample	Sampling date	Mass (g)	Mass concentration (μg/m^3^) over a 24-h period		
			TSP	PM_10_	PM_2.5_
AD-1	3/31/2007-4/2/2007	4.68	82.5–1040.1	57.3–450.0	16.6–70.4
AD-3	5/25/2007-5/27/2007	5.63	280.3–321.6	191.5–203.3	23.6–29.0
NAD-4	5/22/2007-5/23/2007	1.09	90.8	69.5	28.7
NAD-7	12/26/2007-12/27/2007	1.05	49.0	38.1	15.0
NAD-26	11/12/2008-11/14/2008	1.94	82.0	59.6	19.3
