# Supplementary material for: Lead chromate detected as a source of atmospheric Pb and Cr (VI) pollution
Source: Sci Rep. 2016 Oct 25;6:36088. doi: 10.1038/srep36088 (PMC5078766; doi:10.1038/srep36088)
Supplement: Supplementary Information [file srep36088-s1.doc]

Lead chromate detected as a source of atmospheric Pb and Cr (VI) pollution

Pyeong-Koo Lee ^1^, Soonyoung Yu ^2*^, Hye Jung Chang^3^, Hye Young Cho^3^, Min-Ju Kang^4^, Byung-Gon Chae^1^

**Affiliations:**

^1^Geologic Environment Division, Korea Institute of Geoscience and Mineral Resources, 30 Kajung-dong, Yusung-gu, Daejeon, 305-350, Korea.

^2^Korea CO2 Storage Environmental Management Research Center, Korea University, Seoul, 02841, Korea.

^3^Advanced Analysis Center, Korea Institute of Science and Technology, 5 Hwarang-ro 14-gil, Seongbuk-gu, Seoul, 136-791, Korea

^4^Department of soil and Groundwater, Korea Environment Corporation, 42 Hwangyeong-Ro, Seo-gu, Incheon 404-708, Korea

*Correspondence to: iamysy@korea.ac.kr


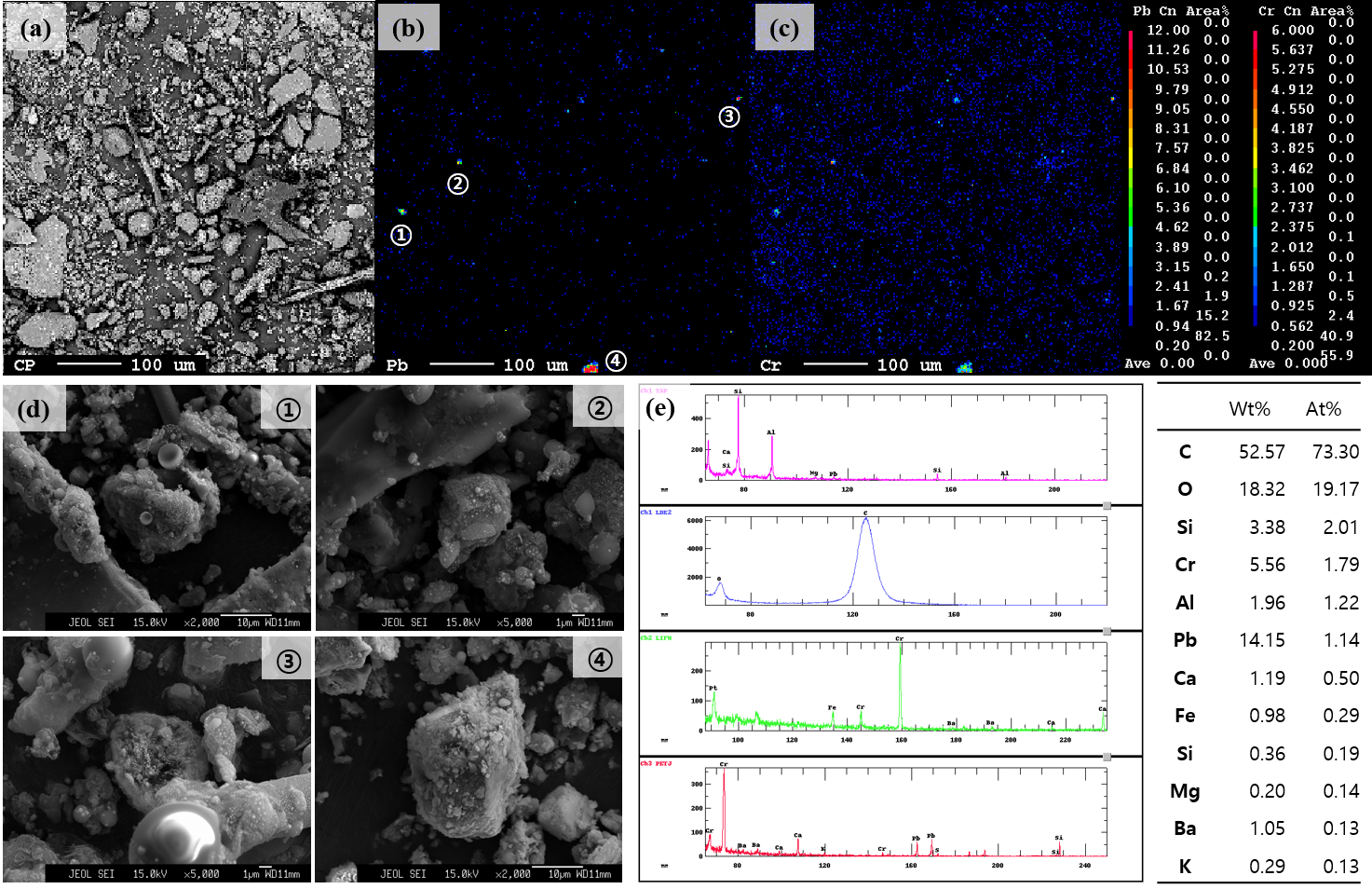


**Supplementary Figure 1:** Occurrence of Pb-Cr-containing particles examined by EPMA-WDS elemental mapping. (a) Image of a dust dry deposition sample (NAD-7) within the scanning area of 585 μm × 585 μm, (b) WDS element map for Pb, (c) WDS element map for Cr, (d) Four Pb-Cr-containing particles magnified, (e) Chemical composition analysis of WDS spectrum for a Pb-Cr-containing particle. Pb in (b) and Cr in (c) were detected together in 9 particles of thousands of particles in (a), 4 particles were magnified in (d), and a representative particle was used for the chemical composition analysis in (e). TAP, LDE2, LIFH and PETJ crystals were used for the elemental detection in (e).

**Supplementary Figure 2:** Surface particles. (a) HAADF STEM image; (b)~(e) EDS element maps showing various minerals such as rutile, quartz, iron oxides, calcite and clay minerals on the surface.

**Supplementary Figure 3:** SEM analysis of a Ti-containing particle in a dry deposition sample (NAD-7). (a) Cross-sectioned SEM image after ion milling using FIB (focused ion beam), (b) HAADF STEM image.

**Supplementary Figure 4:** STEM-EDS element map images of a Ti-containing particle in a dry deposition sample (NAD-7). (a) Merged EDS element map; (b)~(h) EDS individual element maps of C, Fe, Al, O, Ti, Mg and Si.

**Supplementary Figure 5:** Selected area electron diffraction patterns (SADP). (a) TiO_2_ (rutile; tetragonal, P42/mnm(136)), (b) CaMg(CO_3_)_2_ (dolomite; hexagonal, R-3(148), (c) CaO (quick lime, cubic, Fm-3m(225)), (d) SiO_2_ (quartz; hexagonal(primitive)).

**Materials and Methods:**

The dry deposition of AD (N = 2) and NAD (N = 3) were sampled from 31 March 2007 to 14 November 2008 in northwestern Daejeon (36°20’N and 127°22’E) to characterize the nature of pollutants^1,7^. A total of 300 medium-sized stainless steel trays (0.43 x 0.32 m, 5 cm deep) were used to obtain adequate quantities for mineralogical studies. The meteorological conditions in Daejeon are typically influenced by the East Asian monsoon from China. The average wind speed is 1.9 m/s.

Among the dust powders, Pb-containing aggregates were identified based on the morphology and chemical composition using energy dispersive spectroscope (EDS; HORIBA; EMAX x-act), which is embedded in a dual beam focused ion beam (DB-FIB; FEI; Helios Nano-Lab 600). In addition, electron probe micro analyzer-wavelength dispersive spectroscopy (EPMA-WDS; JEOL; JXA-8500F) with field emission gun was used to analyze the occurrence of Pb-Cr-containing particles in the dust sample. EPMA-WDS has higher detection sensitivity than EDS of which the detection limit is about 1000~3000 ppm. For the phase identification of each particle embedded in the dust, selected area electron diffraction patterns (SADP) and EDS elemental maps were acquired using Talos transmission electron microscope (TEM) (FEI; Talos F200X) operated at 200 keV and equipped with a high-brightness Schottky field emission electron source (X-FEG) and Super-X EDS detector system (Bruker; Super-X™). The TEM samples were prepared by FIB with protection layers of epoxy and Pt coated on the sample surface.

Pb-Cr nanoparticles were separated in the TEM HAADF image using the PhotoshopCS6 (Adobe, USA) layer function. The number of particles and the size and area measurements of the carbon matrix and Pb-Cr compounds were performed using the Image J software (National Institute of Health, USA).
